# Efficient precision editing of endogenous *Chlamydomonas reinhardtii* genes with CRISPR-Cas

**DOI:** 10.1016/j.crmeth.2023.100562

**Published:** 2023-08-22

**Authors:** Adrian Pascal Nievergelt, Dennis Ray Diener, Aliona Bogdanova, Thomas Brown, Gaia Pigino

**Affiliations:** 1 Max Planck Institute of Molecular Cell Biology and Genetics, Pfotenhauerstraße 108, 01307 Dresden, Germany; 2 Human Technopole, V.le Rita Levi-Montalcini, 1, 20017 Milan, Italy; 3 DRESDEN-concept Genome Center (DcGC), Center for Molecular and Cellular Bioengineering, Technische Universität Dresden, Dresden, Germany

**Keywords:** plant biotechnology, Chlamydomonas, CRISPR-Cas9, genome editing, endogenous tagging, high-throughput screening

## Abstract

CRISPR-Cas genome engineering in the unicellular green algal model *Chlamydomonas reinhardtii* has until now been primarily applied to targeted gene disruption, whereas scarless knockin transgenesis has generally been considered difficult in practice. We have developed an efficient homology-directed method for knockin mutagenesis in Chlamydomonas by delivering CRISPR-Cas ribonucleoproteins and a linear double-stranded DNA (dsDNA) donor into cells by electroporation. Our method allows scarless integration of fusion tags and sequence modifications of proteins without the need for a preceding mutant line. We also present methods for high-throughput crossing of transformants and a custom quantitative PCR (qPCR)-based high-throughput screening of mutants as well as meiotic progeny. We demonstrate how to use this pipeline to facilitate the generation of mutant lines without residual selectable markers by co-targeted insertion. Finally, we describe how insertional cassettes can be erroneously mutated during insertion and suggest strategies to select for lines that are modified as designed.

## Highlights

• CRISPR-Cas9 allows endogenous tagging of *Chlamydomonas* genes
• High-throughput crossing reduces the time to combine mutant traits
• An optimized PCR/qPCR protocol allows robust genotyping of crude extracts
• Insertional mutagenesis is impeded by microhomology-driven fragment exchange

## Motivation

The ability to perform precise genome editing is an indispensable tool in the study of any model organism. In *Chlamydomonas*, a long-established model organism for study of genetics, cilia motility, and photosynthesis, homology-directed precision editing has been considered difficult. Here, we present a CRISPR-Cas ribonucleoprotein-based method for homology-directed knockin mutagenesis of *Chlamydomonas* and demonstrate that the approach can produce endogenously tagged cell lines in a matter of weeks.

## Graphical abstract

**Figure:**
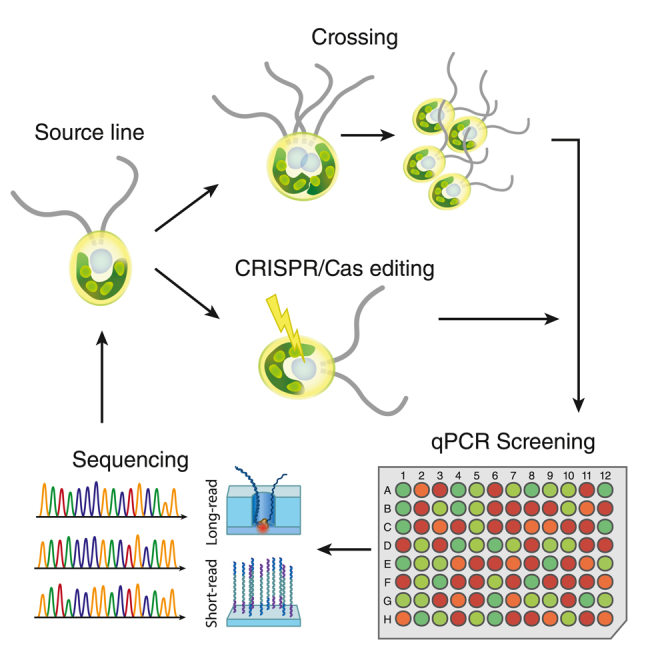

## Teaser

Nievergelt et al. present a CRISPR-Cas-mediated knockin approach for precision genome editing in *Chlamydomonas*. The work also generates rapid crossing and robust high-throughput genomic screening strategies and provides a toolbox for the Chlamydomonas community to rapidly produce the precise mutant line required for any given study.

## Introduction

The green unicellular alga *Chlamydomonas reinhardtii* is a popular model organism for topics ranging from structure and function of cilia and basal bodies^1^^,^^2^ and chloroplast biogenesis^3^ and photosynthesis^4^ to circadian rhythm^5^ and shares many protein homologues with higher eukaryotes.^6^ In practice, *Chlamydomonas* offers a number of highly desirable traits for experimental work^7^: cells grow readily by vegetative division in minimal media, both in suspension as well as on solid media, allowing for the generation of large biomass as well as simple isolation of clonal cells. Cell lines of opposite mating types can be crossed by sexual reproduction to combine genetic traits.^8^ Additionally, vegetative cell lines are haploid, which facilitates genetic editing, as only one successful alteration of a gene is necessary.^9^ Finally, *Chlamydomonas* tends to integrate exogenous genetic material via random insertion into its genome, primarily by non-homologous end joining (NHEJ). This property has enabled the realization of the Chlamydomonas Library Project (CLiP),^10^ the primary source of insertional mutants for the field.

Traditionally, such mutants are rescued by introduction of a recombinant copy of the disrupted gene. Such rescue experiments also allow for the introduction of fusion tags for biochemical or optical interrogation. However, rescue experiments become increasingly difficult for larger genes and will additionally disrupt a new, random, and thus unknown part of the genome. Pioneering works by Greiner,^11^^,^^12^ Shin,^13^ and Picariello^14^ have established targeted insertion of exogenous DNA by the CRISPR-Cas9 system^15^ for gene disruption in *Chlamydomonas*. Additionally, Greiner^11^ demonstrated a successful mCherry fusion to Channelrhodopsin1 by homology-directed repair (HDR) using zinc finger nucleases. Successful attempts at functional knockin repair of short pieces using single-stranded DNA templates co-transformed with large amounts of Cpf1 have been reported with potentially high integration rates, with, however, a very high variability for different loci^16^ (see Table S1). Single-stranded donor templates are limited in size and are prone to form secondary structures, which may inhibit efficient transformation for some designs.

We have developed a method for homology-directed knockin mutagenesis suitable for large functional inserts of several kilobases by fusing a functional insert to a resistance cassette. A similar approach has independently been shown by Hou et al.^17^ Here, we expand this technology by demonstrating efficient and robust endogenous tagging of *Chlamydomonas* genes with and without fused cassettes and showing how tags can be serially introduced or combined by meiotic crossing as well as how to robustly and time efficiently screen and verify the resulting recombinant lines.

## Results

### CRISPR-Cas-based precision mutagenesis

The CRISPR-Cas system can create frameshift-induced knockouts in most model organisms due to imperfections in DNA repair and the resulting scars at the cut site.^18^ *Chlamydomonas*, however, has evolved an unusually efficient DNA-repair system,^19^ most likely a consequence of being a haploid organism that prefers exposure to sunlight, which is accompanied by significant doses of harmful UV radiation. As such, when creating mutant lines in *Chlamydomonas*, it is important to transform cells with a piece of donor DNA (dDNA), which is to be introduced into the cut site. The dDNA typically contains a resistance cassette to select for successful transformants. While it is possible to insert the dDNA via NHEJ, it is preferable to insert dDNA via HDR to control orientation and preserve reading frames. To leverage HDR, the dDNA must be flanked on either side by regions with homology to the genomic region up- and downstream of the cut site. These homology arms are ideally about 50 bp in length.^14^

**Figure 1:**
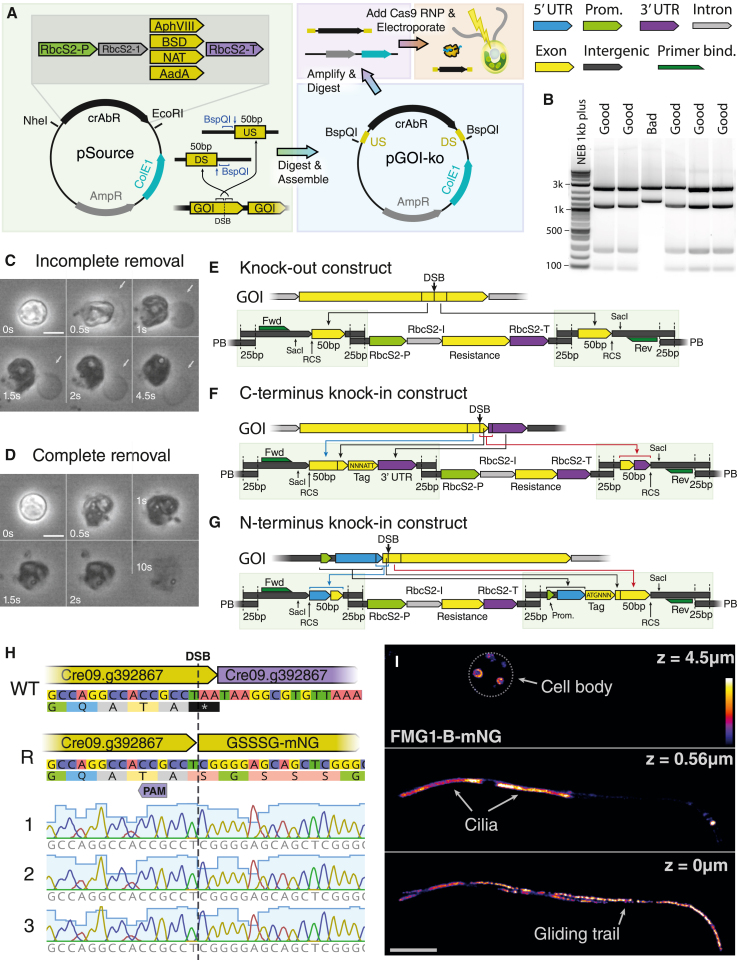
*Chlamydomonas* CRISPR-Cas mutagenesis and design of dDNA (A) Overview of the mutagenesis process: base resistance vectors are double digested, and homology arms upstream (US) and downstream (DS) of the double-stranded break (DSB) on a gene of interest (GOI) are added with flanking remote cutting restriction sites (RCSs) as synthetic fragments by Gibson assembly to a *Chlamydomonas* antibiotic resistance gene (crAbR). The resulting knockout vector is amplified, digested with the corresponding type IIs enzyme, purified, and transformed into wall-stripped cells by electroporation together with Cas9 ribonucleoproteins (RNPs). (B) Analytical SacI restriction sites allow for easy identification of good or bad bacterial clones. (C) Insufficiently digested cell walls remain visible after lysis with detergent (white arrow). Scale bar: 5 μm. (D) Cells with fully digested cell walls dissolve completely within a few seconds after lysis initiates. Scale bar: 5 μm. (E) Knockout cassette design: synthetic linkers (green boxes) containing 50 bp homology arms consisting of genomic sequences US and DS of the DSB, primer binding sites, analytical (SacI) and preparatory RCS are assembled by 25 bp overlaps to an antibiotic resistance cassette and to a plasmid backbone (PB) by homology cloning. The cABr cassette consists of a resistance gene under control of the rubisco promoter (RbcS2-P) terminator (RbcS2-T) pair. (F) For C-terminal knockin tags, the linker US of the antibiotics cassette is extended with the remainder of the C-terminus after the DSB, followed by the desired tag/stop codon and the cloned 3′ UTR. (G) In analogy to (F), N-terminal knockin tags are constructed with the DS linker extended with the endogenous 5′ UTR, followed by ATG and the desired tag and the endogenous N terminus up to the DSB before the 50 bp homology arm. (H) Representative Sanger sequencing traces showing repeatable scarless homology-directed integration of the GS_3_G-mNeonGreen tag at the C terminus of FMG1-B. See also Table S1. (I) Spinning disk confocal sections of FMG1-B-mNeonGreen cells, showing brightly labeled cilia (middle), trails of FMG1B-mNG due to gliding on the glass slide (bottom), and fluorescent intracellular vesicles (top). Scale bar: 5 μm.

Based on the previous methodological developments,^14^ we have established CRISPR-Cas9-based gene disruption as a first step. To this end, we have constructed a set of vectors that allow for easy assembly of insertional cassettes by adding homology arms to an insert containing a selectable marker by Gibson assembly. We use resistance markers to paromomycin,^20^ nourseothricin,^21^ blasticidin S,^22^ and spectinomycin^23^ under the control of the RbcS2-promotor/RbcS2-1 intron/RbcS2-terminator regulatory elements (Figure 1A). In addition to homology arms, we introduce artificial primer sites, diagnostic restriction sites (Figure 1B), and, importantly, remote cutting restriction sites that allow for precise excision of the dDNA from the backbone. The final constructs are digested with type IIs restriction endonucleases, purified, and delivered together with Cas9 ribonucleoprotein (RNP) by electroporation into heat-shocked cells stripped of their cell walls with autolysin (Figure 1A).

In our quest to increase the insertional efficiency, we have identified two key parameters: complete cell wall removal (Figure 1C and 1D) and the purity of the transformation reagents (see STAR Methods). Importantly, we have found that the purity of the DNA used for transformation is one of the most common reasons for failed transformations and that the kit used for preparation of the DNA is crucial (see STAR Methods). We further reduce endotoxins in the purified DNA by an additional column-purification step. The requirement for highly pure dDNA is well established in other eukaryotic systems.^24^ Additionally, we find that the transformation procedure is sensitive to a number of key parameters, most notably the concentrations of dDNA and RNP, the density of cells during heat-shock treatment, and the electroporation system used (see STAR Methods). In our hands, the fully optimized process results in, on average, about 63% (and up to 90%) of the colonies expressing the selectable marker, with inserts at the location of the intended double-stranded break (DSB) induced by Cas9 (Figure S1; Table S1).

Importantly, the quantification of the lengths of the resulting insertions by PCR generally exhibit one discrete size that appears more frequently than others (see also Figure 4C), indicating a favored outcome of an insertion experiment. Sanger sequencing of these insertions reveals almost all of these to be the result of perfect HDR (see Table S1). We thus reasoned that this methodology, which has been primarily used for gene disruption (Figure 1E) in *Chlamydomonas*, can be directly extended to creating functional knockin edits at endogenous loci.

Using our resistance marker constructs, we extended the flank upstream of the cassette in addition to a 50 bp homology arm by the short sequence starting at the DSB, up to (but not including) the stop codon, sequence coding for a fusion tag with linker, a full clone of the endogenous 3′ UTR of the gene of interest to create a C-terminal (cTer) tagging construct (Figure 1F). Similarly, an N-terminal (nTer) knockin construct can be assembled by extending the downstream homology arm by the endogenous 5′ UTR and a desired tag (Figure 1G).

As proof of principle, we chose to fuse a fluorescent mNeonGreen tag with a GS_3_G linker to the cTer of the flagellar major glycoprotein FMG1-B (Cre09.g392867v5).^25^ We chose a cut site close to the cTer of the coding sequence and designed a corresponding dDNA vector. When digested and electroporated into cells together with the corresponding Cas9 RNP and selected on antibiotics, this construct resulted in colonies, of which 12/48 exhibited identical scarless repair as designed (Figure 1H; Table S1). Importantly, the majority (9/12) of these colonies consisted of cells with bright green fluorescent flagella, as expected of a fluorescently labeled flagellar coat protein (Figure 1I; Video S1).

**Figure 2:**
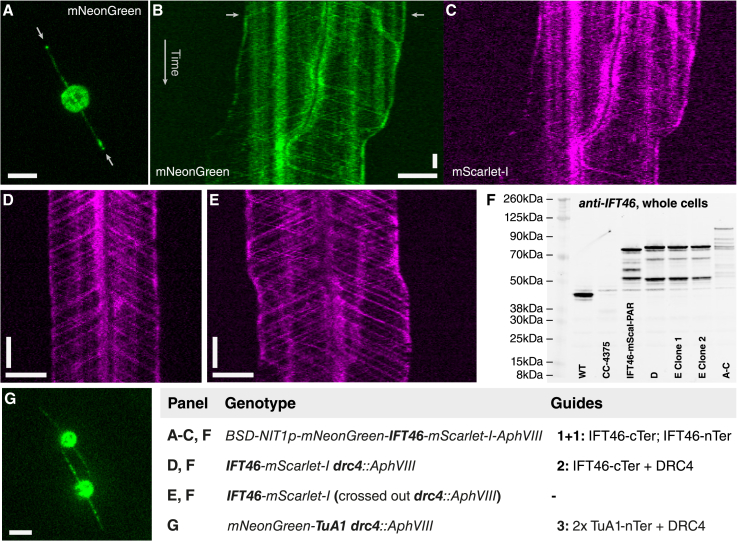
CRISPR-Cas editing allows for advanced genetic designs (A–C) Maximum intensity projection of *BSD-mNeonGreen-IFT46-mScarlet-I-AphVIII* serial dual-knockin construct (white arrows indicate ciliary tips) (A) with corresponding kymograms (B and C) showing co-localized intraflagellar transport (IFT) signals. (D) IFT kymogram of *IFT46-mScarletI DRC4::PAR* co-targeted line with paralyzed flagella (see also Data S2). (E) IFT kymogram of marker-free progeny of *IFT46-mScarletI DRC4::AphVIII* obtained by back-crossing. (F) Immunoblot of different IFT46 lines showing complete band upshifts as well as truncation/degradation and expression-level changes in whole-cell lysates (see Data S2 for total protein). (G) Maximum intensity projection of endogenous *mNeonGreen-TuA1 DRC4::AphVIII* co-targeting construct generated by multiguide fragment substitution. See also Figure S2. All horizontal scale bars represent 5 μm, and all vertical scale bars represent 10 s.

Video S1. Fluorescence wide-field timelapse of FMG1-B-mNeonGreen fusion knockin cells shows intracellular as well as ciliary fluorescence in swimming cells, related to Figure 2

In addition to fluorescent cilia (Figure 1I, middle panel), these cells can deposit bright trails of fluorescent proteins (Figure 1I, bottom panel), most likely in small ectosomes^26^ bound to the glass surface. Finally, we observe intracellular localization of the protein to large bubble-like structures (Figure 1I, top panel). This result demonstrates how HDR-driven endogenous knockins in *Chlamydomonas* can be used to generate fusion proteins. We have validated this method of editing for tagging at both termini with multiple different fluorescent tags, even in combination (see Figures 2A–2C and 2F).

Finally, this can be extended to a co-targeting approach to allow for the generation of mutant lines with almost any genetic modification without residual resistance markers: the intended insertion is targeted by homology to a DSB (Figures 2D and 2F) or a multi-RNP excision and replacement (Figure 2G) while a second co-transformed antibiotic cassette insertion is targeted to a gene, which results in an observable phenotype such as paralyzed flagella (e.g., DRC4 or ARMC2). While the secondary insertion is present in most resistant colonies, correct insertion of the primary target is typically present in only a few percent of resistant colonies, necessitating extra screening. The secondary insertion is used for antibiotic selection and screening and can later be crossed out to generate a final modified *Chlamydomonas* line free of selective markers (Figure S2).

### Rapid crossing using fluorescence-activated cell sorting (FACS)

**Figure 3:**
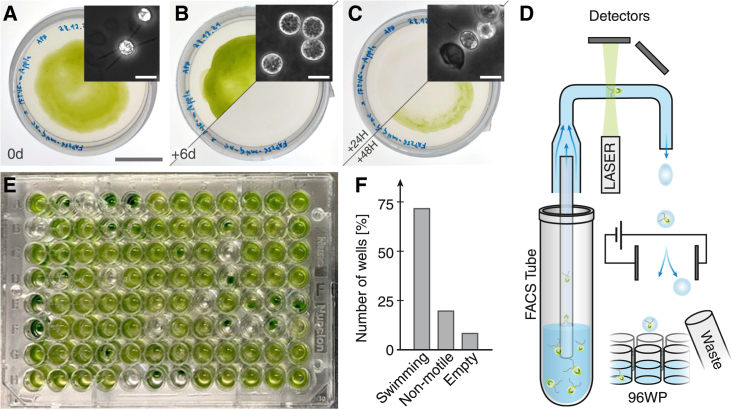
Crossing cell lines with FACS separation allows for combining genetic traits in minimal time (A) Cells of opposite mating type are converted to gametes and mixed to obtain quadroflagellates (see inset), then plated on TAP agar. Scale bar: 20 mm; inset scale bars (A–C): 20 μm. (B) After 6+ days in the dark, plates are washed to remove vegetative cells, leaving behind a layer of mature zygospores (see inset). (C) One day after removing the vegetative cells, zygospores hatch, leaving behind the sporangial walls (see inset). A light green film of progeny is visible on plates. (D) Hatched progeny are suspended in medium and distributed by FACS as single cells into a 96-well plate while size selecting by laser forward-scattering/side-scattering profile. (E) Example plate of progeny from *FAP256-mNeonGreen* crossed to *ift46::NIT IFT46-mApple*, 1 week after sorting. (F) The majority of the wells (∼73%) shown in (E) contain swimming cells, ∼20% are non-motile (visible as clumps of cells), and ∼8% of wells are empty.

Meiosis in *Chlamydomonas* is commonly used to combine genetic traits or to back-cross mutant lines to a wild type to remove unwanted secondary mutations.^8^ Under nitrogen starvation, *Chlamydomonas* cells convert into gametes, which can fuse with gametes of the opposite mating type. The resulting dikaryotic cells (Figure 3A) then form a zygospore (Figure 3B), which, after maturation and subsequent exposure to light, hatches into four daughter cells of distinct genetic makeup (Figure 3C). Traditionally, the hatched tetrad of cells are separated on a per-zygospore basis by hand on an agar plate before analyzing the resulting cells for genetic segregation.^8^^,^^9^ However, the process of moving individual cells by hand is difficult and lengthy and often results in contaminated plates. The emergence of affordable whole-genome sequencing has somewhat reduced the importance of tetrad dissection, especially for endogenous edits. In an effort to facilitate and speed up the crossing process, we use FACS instrumentation to rapidly separate hatched cells into 96-well plates (Figure 3D).

Specifically, cells are pumped from a liquid suspension into a fluidic system where a sheath flow spatially separates the cells, which are then measured for scattering and fluorescent properties in a laser optical system before they are cast into droplets that are individually manipulated. While FACS has been used to sort cells based on fluorescence,^27^ most proteins, when tagged endogenously, are too low in abundance to be detectable over the autofluorescent background of *Chlamydomonas*. Thus, we use FACS to rapidly and conveniently separate freshly hatched cells that have been washed off an agar plate into 96-well plates, one single cell per well, regardless of the fluorescence signal (Figure 3E). As a benchmark, we mated the CRISPR-Cas knockin line *FAP256-mNeonGreen* to the fluorescent intraflagellar transport line *ift46::NIT IFT46-mApple* created by insertional rescue. While occasional empty wells can be found, we have, in hundreds of wells tested for mating type, never observed a well with a mixed genotype resulting from two cells being sorted into the same well. The resulting phenotypes observed in the wells of the sorted plate match the roughly 1/4 probability of a non-motile cell line (Figure 3F). As such, the well plate can be directly used for high-throughput genotyping as soon as the cells have divided to a usable density.

### High-throughput screening by qPCR

Having established the feasibility of knockin fusions, we realized that the limiting factor for creating cell lines with endogenous tags is the selection of the correct colonies and set out to remove this bottleneck by developing a highly robust screening procedure based on quantitative PCR (qPCR). A qPCR-based approach is high-throughput compatible and drastically reduces the number of DNA gel electrophoresis steps: inserts can be directly detected based on amplification curves, and HDR inserts can be identified by high-resolution melting analysis of the products. The resulting candidates can then be verified by Sanger sequencing and phenotypic analysis. However, qPCR is sensitive to PCR inhibitors found in crude cell lysate, and we have found multiple commercially available polymerases to be unable to amplify even short amplicons. However, it is crucial to be able to use crude lysate as a template for genotyping PCR, as purification is prohibitively work intensive.

**Figure 4:**
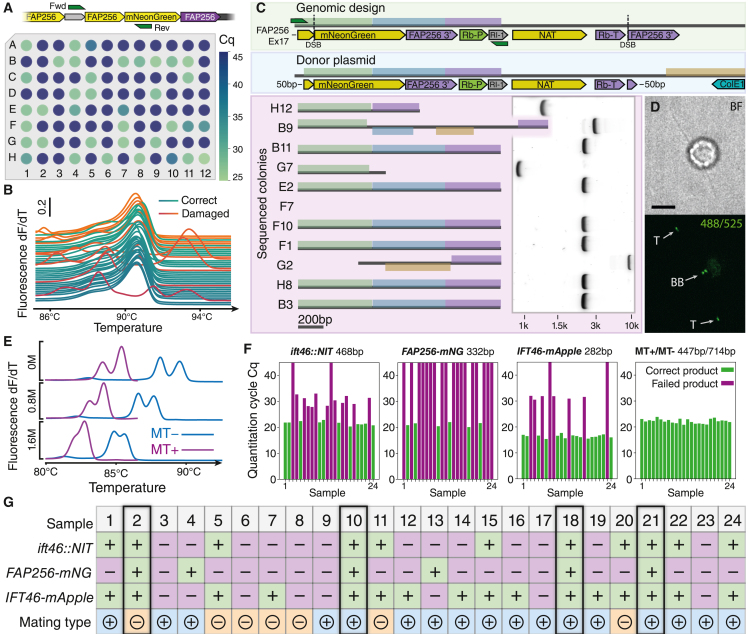
High-throughput screening allows rapid pre-selection of CRISPR-Cas-mediated knockin colonies with homology-directed repair insertions as well as full genotyping of subsequent crossing products (A) Quantitation cycle (C_q_)-based screening for insertion by primers flanking the intended insertional junction. Colonies with a low C_q_ (green) are likely to have the insert. See also Figure S3. (B) High-resolution melting (HRM) analysis of the products of (A) allows for direct classification into wanted homology-directed repair insertions (green curves) and unwanted incorrect insertions (red curves). (C) Simplified Mauve^29^ alignment of insertion sequences of 10 colonies selected based on HRM analysis in (B) to the genomic design and the donor plasmid used to generate the strains. Large-scale rearrangements with the donor plasmid are seen in clones B9 and G2. Clones B11, E2, F10, F1, H8, and B3 are identical and exhibit a genotype as designed. Amplicons with indicated primers (green) used for sequencing shown in agarose gel electrophoresis (see also Data S2 and Figure S4). (D) Bright-field (top) and fluorescence (bottom) micrographs of a ciliated *FAP256-mNeonGreen* (*B11*) knockin cell confirm the expected localization at the ciliary tip (T) and the basal body (BB). Scale bar: 5 μm. (E) Mating types can be differentiated by high-resolution melting analysis of qPCR products. (F) Absolute C_q_ analysis allows direct genotyping of crossing products between the *FAP256-mNeonGreen* knockin line B11 (A–D) and a fluorescently marked IFT line (*ift46::NIT IFT46-mApple*; see Figure 4) by genotype-specific primers. (G) Based on the genotypes identified in the qPCR measurement in (F) together with mating type determination as in (E), the cell line required for DS processing can be directly selected without further analysis. Double-fluorescent cells are highlighted by black borders.

We have tested multiple modern engineered polymerases to find conditions that allow for almost plate-level reliable amplification of large amplicons from crude lysate. We performed qPCR amplification of four genes of different sizes from crude template using five different polymerases (Toyobo KOD One, Takara PrimeStar GLX, NEB Q5, Invitrogen Platinum SuperFi2, and Invitrogen Platinum2 Taq) with different GC enhancers (as-is, 0.8 M betaine, 1.6 M betaine, 0.8 M betaine + 0.81 M propylene glycol^28^). We have found KOD One and Platinum SuperFi2 polymerases to provide the most robust amplification of even long amplicons, followed by PrimeStar GLX and Q5, which are significantly inhibited by crude lysate (Figure S3). Platinum 2 Taq resulted in robust amplification of shorter fragments from crude lysates but, as a non-processive polymerase, is less suited to longer fragments. The addition of 0.8 M betaine to the qPCR reaction is generally beneficial and resulted in improved specificity (Figure S3). In our hands, KOD One polymerase has an undesired tendency to yield non-specific amplification, especially in the absence of the desired amplicon in the template. Finally, of the evaluated polymerases only Platinum SuperFi2 and Platinum 2 Taq resulted in clean high-resolution melting (HRM) profiles. As such, we base our screening procedure on a commercial low-inhibition qPCR dye used with Platinum 2 Taq for smaller pieces (<2 kB) and with SuperFi2 for larger fragments. Using this custom master mix, we are able to robustly amplify the flanks of insertional mutants at the plate level (Figure 4A; Data S2), suitable for post-PCR HRM analysis of the products (Figure 4B; see also the STAR Methods).

Strikingly, different colonies that have integrated a fluorescent fusion knockin by HDR do not invariably exhibit the expected fluorescence signal. In addition to integrating the intended cassette, we find that these cases also exchange fragments of the non-selective part of the insert by recombination of microhomology domains, thus rendering the insert non-functional (Figure 4C; Data S2). In contrast, correct clones all exhibit the expected fluorescent signal (Figure 4D). We recommend verifying the insertions of all new cell lines by Sanger sequencing.

Aside from defects during integration of the dDNA, CRISPR-Cas9 is known to cause off-target defects.^30^ Additionally, *Chlamydomonas* can integrate parts of the dDNA in non-intended loci of the genome.^10^ To search for these unintended changes and gauge the frequency at which this happens in fusion knockin lines, we performed whole-genome sequencing by short reads on four single knockins as well as one double-knockout line and compared the resulting assemblies to the background genome. While we did not detect any off-target effects caused by Cas9, we have found random non-homologous integration events of vector fragments, as is typical of *Chlamydomonas*, in one knockin line as well as in the double-knockout line (Figure S4). This result is consistent with the ∼10%–20% of resistant colonies that do not have an insertion at the DSB and that must have inserted the resistance by random insertion. Thankfully, such unwanted insertions are often not problematic and can be easily removed by back-crossing to the wild type if so desired.

In addition to pre-screening CRISPR-Cas lines, our qPCR approach is directly applicable to genotyping meiotic progeny. By assembling a reaction with two primer pairs, each specific to one mating type, and amplifying products with different melting temperatures, the mating type can be directly determined by HRM (Figure 4E). Additionally, each trait can be queried by a specific qPCR reaction (Figure 4F). Finally, the cell lines that contain the desired combination (Figure 4G) and mating type for downstream processing can be selected and propagated without further need for slab-gel analysis or sequencing.

## Discussion

*Chlamydomonas* has, for a long time, been considered a comparatively difficult organism in terms of genetics in particular and genetic engineering specifically. While integration by HDR without CRISPR-Cas is common practice in organisms such as yeast,^31^ *Chlamydomonas* requires a genomic DSB and a linearized donor fragment for integration by HDR, which has historically been an impediment to the wider adoption of *Chlamydomonas* as a model. In addition to this, the average 64% GC content of the genome, which can rise to over 90% for islands of significant size, has made practical work with *Chlamydomonas* genes challenging since traditional polymerases tend to stall or stop when encountering strong secondary structures typically found in such regions.^32^ The methods described in this work offer solutions to both of these problems. The use of the CRISPR-Cas systems is ubiquitous in modern research, and all required resources are available at high purity from commercial sources. Likewise, the polymerases and additives that have been evaluated here are readily available and have, in our hands, been able to amplify even long pieces (>10 kBp) of genomic *Chlamydomonas* DNA directly from purified or even crude cell lysate using QuickExtract (Lucigen) solution. As such, even difficult-to-clone genes can be studied without major difficulties. Finally, compared with traditional rescue mutagenesis, CRISPR-Cas-based mutagenesis does not require a full clone of the coding sequence of the gene and thus uses much shorter dDNA sequences, which significantly simplifies cloning and improves transformation efficiency in *Chlamydomonas*.

The method here has proven to work robustly well in multiple labs. On the other hand, reports of low integration efficiency for CRISPR-Cas experiments are common. In our experience, many of these failed attempts can be traced back to impure reagents, in particular the quality of the dDNA used. Prominently, the widespread Qiagen column plasmid isolation systems have yielded very poor integration rates. Possible reasons for this are contamination of the final product with chaotropic salts or bacterial components such as lipopolysaccharides. Similar problems are observed with certain batches of homemade Cas9. As salt contamination is generally not an issue with lab-purified proteins, we reason that residuals of bacterial components are likely the cause of poor mutagenesis. We believe that most modern column-based plasmid isolation systems can be used as long as they are designed for obtaining highly pure DNA intended for use in sensitive systems such as primary cell lines or immune cells.

A potential drawback of fusing the antibiotic resistance cassette and corresponding regulatory elements directly to the insertion is that the resistance can no longer be crossed out. Since even with modern electroporation systems the absolute transformation efficiency remains between 10^−4^ and 10^−5^, selectable markers will remain necessary until higher efficiencies can be reached. While in most cases the presence of the resistance is unproblematic, the presented advanced designs that make use of multiple guides to co-target multiple loci are an elegant way around the problem, albeit at increased screening effort, since the efficiency of non-selected-for inserts drops to a few percent. This lower efficiency can be partially counteracted by adjusting the concentration of the non-selectable inserts with respect to the selectable ones. Another potential solution is to excise an extended resistance cassette in a mutant line with a verified correct integration using the Cre/*loxP* system.^33^

Finally, endogenous editing makes it possible to make precision changes without disturbing the genomic environment such as chromatin conformation, which is known to regulate gene expression. On the other hand, we show here that expression levels can be significantly impacted by introducing fusion cassettes (Figure 2F), especially when exchanging promoters, and that protein fusions can lead to truncations or degradations not present in the wild type.

In conclusion, we show that knockin constructs for endogenous tagging are not only possible but are a feasible route to genetic engineering in *Chlamydomonas*. We believe that our approach has significant advantages over traditional rescue-type constructs, such as single-copy integration, and offers the possibility of leaving edited genes under the control of their endogenous transcriptional elements. We expect this method will find widespread use in the field to accelerate and enable work with this easy-to-culture and versatile model organism. We hope that our developments will be enabling both on a local lab level as well as on a community level, with the potential to generate genome-wide tagged libraries and to lead to a wider adoption of *Chlamydomonas*.

### Limitations of the study

The methods presented here allow for near-arbitrary genomic changes, but it has to be noted that a successful genetic edit can still result in a non-functional protein product or expression level changes due to misfolding or changes to regulatory elements. Editing lethal genes in *Chlamydomonas* could result in no or lower progeny, and we have not directly targeted any such genes in this work. Additionally, we have not targeted chloroplast or mitochondrial genes with our methods.

## STAR★Methods

### Key resources table

**Table:** 

REAGENT or RESOURCE	SOURCE	IDENTIFIER
**Antibodies**		

anti-IFT46	Rosenbaum lab	17600
IRDye 800CW Goat anti-Rabbit IgG	Li-Cor	926–32211

**Bacterial and virus strains**		

E. Coli Dh5α	NEB	Cat#C2987H
E.Coli GB06	Lab stock	N/A

**Chemicals, peptides, and recombinant proteins**		

Alt-R® S.p. Cas9 Nuclease V3	IDT	Cat#1081058
Platinum™ SuperFi II PCR Master Mix (2x)	Thermo Fischer	Cat#12368010
Platinum™ II Hot-Start PCR Master Mix (2X)	Thermo Fischer	Cat#14000013
Paromomycin	TCI	P2092
Nourseothricin	Jena Bioscience	AB-102XL
Blasticidin S	Carl Roth	CP14.2
Spectinomycin	Merck	S4014-5G
EvaGreen Plus	Biotinum	31077-T
GelGreen	Biotinum	41005
Betaine	Merck	61962
NEBuilder HiFi assembly MM	NEB	E2621L
BspQI	NEB	R0712L
SacI-HF	NEB	R3156L
EcoRI-HF	NEB	R3101L
NheI-HF	NEB	R3131S
Hutner’s Trace Elements	Chlamydomonas Resource Center	N/A
IGEPAL CA-630	Merck	I8896

**Critical commercial assays**		

ZymoPURE II Plasmid Midiprep Kit	Zymo Research	D4200
ZymoPURE II Plasmid Miniprep Kit	Zymo Research	D4211
DNA Clean & Concentrator-25	Zymo Research	D4033
Neon electroporator	Thermo Fisher	N/A
10μL Neon transfection kit	Thermo Fisher	MPK1025
QuantStudio 7 Pro	Thermo Fisher	N/A
Light board	Artograph	LightPad 940LX
FACS sorter	Sony	MA-900
iBlot2	Thermo Fisher	N/A
iBlot2 PVDF stacks	Thermo Fisher	IB24002
iBind Flex	Thermo Fisher	N/A
Odyssey	LiCor	N/A

**Experimental models: Organisms/strains**		

Chlamydomonas WT (+)	Chlamydomonas Resource Center	CC-620
Chlamydomonas WT (−)	Chlamydomonas Resource Center	CC-621
Chlamydomonas WT (−)	Dutcher Lab	CC124 32M
Chlamydomonas WT (+)	Chlamydomonas Resource Center	CC-125
FMG1B-mNeonGreen	This study	CC-6012
mNeonGreen-IFT46-mScarletI	This study	CC-6013
IFT46-mScarletI drc4:AphVIII	This study	CC-6014
IFT46-mScarletI (no resistance markers)	This study	CC-6015
mNeonGreen-TuA1 drc4:AphVIII	This study	CC-6016
FAP256-mNeonGreen	This study	CC-6017
ift46:NIT IFT46-mApple	This study	CC-6018
FAP256-mNG ift46:NIT IFT46-mApple	This study	CC-6019

**Oligonucleotides**		

Alt-R® CRISPR-Cas9 tracrRNA, 20 nmol	IDT	Cat#1072533
Oligonucleotides used in the study	This paper	Data S1.zip/Chlamy CRISPR cas supplemental oligos.xlsx

**Recombinant DNA**		

pAB262_NAT-R	This study	N/A
pAPN_BSD	This study	N/A
pAPN_AphVIII	This study	N/A
pAPN_AadA	This study	N/A
pE345	Unknown	N/A
pALM32	Chlamydomnas Resource Center	N/A
CRISPR donor vectors generated in the study	This study	Data S1.zip/Chlamy CRISPR cas supplemental oligos.xlsx
Insertional donor vectors generated in the study	This study	Data S1.zip/Chlamy CRISPR cas supplemental oligos.xlsx

**Software and algorithms**		

Geneious Prime	Biomatters	2021–2023.0.1
Design and Analysis	Thermo Fisher	2.6.0
Illustrator	Adobe	27
Fiji	Fiji	https://imagej.net/software/fiji/
Photoshop	Adobe	27
iQ3	Andor	https://andor.oxinst.com/products/iq-live-cell-imaging-software/

### Resource availability

#### Lead contact

Further information and requests for resources and reagents should be directed to and will be fulfilled by the lead contact, Gaia Pigino (gaia.pigino@fht.org).

#### Materials availability

Strains and plasmids generated in this study have been deposited to the Chlamydomonas Resource Center. The collection numbers for the strains are listed in the key resources table. These are also available from the lead author upon reasonable request.

### Experimental model and study participant details

#### Chlamydomonas cell culture

We received CC124-32M as a kind gift from Dr. Susan Dutcher. CC-4375 (*ift46-1::NIT1*) was obtained from the Chlamydomonas Resource Center. Cells for this study were cultured on 1.5% TAP agar plates or in liquid TAP medium in 96-well plates or aerated 500 mL bottles. Cells were grown at room temperature without specialised temperature control (∼21°C–25°C). For liquid culture, cells are bubbled with air and illuminated by 14h/10h light-dark timed fluorescent tube lights at 60–330 μmol/m^2^/s. Agar plates are grown in aluminum coated trays under 14h/10h light-dark timed LED lamps at ∼20 μmol/m^2^/s. Cells grown on agar for crossing experiments, cells in 96-well microtiter plates as well as cells undergoing overnight recovery after electroporation are kept on an LED light board which is constantly at (LightPad 940LX) at ∼60 μmol/m^2^/s.

TAP-N for gametogenesis was made by replacing NH_4_Cl with equimolar KCl. IFT46-mApple strain was obtained by transformation of CC-4375 with linearized pIFT46-mApple plasmid. Swimming colonies resulting from selection on paromomycin plates were screened by optical microscopy.

### Method details

#### Resistance cassettes construction and cloning

The nourseothricin resistance cassette was ordered as a gene fragment from Genscript. Blasticidin S resistance was ordered as a gene fragment from Eurofins. AphVIII and AadA genes were amplified from pE345 and pALM32 plasmids, respectively. All resistances were cloned into a high-copy vector containing the RbcS2 promoter and intron as well as the RbcS2 terminator using either restriction cloning or Gibson assembly.

All Gibson reactions were assembled with 2x NEBuilder Hifi assembly master mix and incubated for 30 min at 50°C. Fragments below 200bp were added in 2-fold molar excess. Assembled DNA was transformed into chemically competent *E.coli* (GB06 or Dh5a). Mini-preps from LB liquid cultures were done using ZymoPure MiniPrep kit. Midi-prep for transformation into *Chlamydomonas* was done using ZymoPure MidiPrep kit including EndoZero columns for endotoxin removal. dDNA prepared by MidiPrep was digested with the corresponding restriction enzyme (usually NEB BspQI) according to manufacturer’s instructions and column purified by Zymo Clean and Concentrator 25 kit to ∼400 ng/μL. The IFT46-mApple plasmid was created by replacing the EcoRI-EcoRV YFP containing fragment in pE345 (IFT46-YFP) by an EcoRI-EcoRV fragment encoding the mApple ORF.

**Table:** 

Condition	Colonies/Rxn	# Picked	# Non-swimming	% Efficiency
Zymo Midiprep Zymo DCC-25 - Autolysin (+TAP-N)	23 ± 10	47	8	17%
Zymo Midiprep Zymo DCC-25 + Autolyin	551 ± 91	96	66	68%
Qiagen Midiprep Qiagen PCR cleanup + Autolyin	600 ± 70	96	37	37%

Comparison of the effect of autolysin treatment and different DNA quality on targeted gene disruption by integration of a blasticidin S resistance cassette into exon 12 of IFT140, resulting in non-swimming colonies. All cells used were pre-treated equally except that TAP-N was used instead of autolysin for the sample without cell wall removal. The presence of cell walls significantly reduces the number of colonies as well as the integration efficiency. While colony number doesn’t significantly differ between different DNA qualities, the amount of correct integrations increases with higher purity. All reactions were performed as a single 10μL electroporation reaction.

**Table:** 

Kit	Nanodrop conc.	A260/A280	A230/A280	Qubit Conc.	Qubit/Nanodrop
ZymoPure Midiprep	2438 ng/μL	1.96	2.29	3090	126.7%
Qiagen Midiprep	1086 ng/μL	1.95	2.31	1150	105.8%

Comparison of DNA quality indicators for IFT140-ko1 plasmid prepared by two different midi-prep kits. Values were measured at 1/10 dilution for accuracy and verified on two different Nanodrop instruments.

#### Guide RNA selection and RNP preparation

crRNAs were designed in Geneious Prime and scored for off-targets and activity^30^ by soring against the *Chlamydomonas* genome v5 or v6. Guides were selected to be close to the desired locus, have no detected off targets and ideally have an activity score above 0.5. crRNAs and tracrRNA were purchased from IDT. crRNA and tracrRNA were reconstituted to 100μM in nuclease-free duplex buffer (IDT, 30 mM HEPES pH 7.5, 100 mM potassium acetate). 10μL of crRNA and tracrRNA were mixed, heated to 95°C for 2 min and then removed from the heat block to cool to room temperature (RT) to allow the formation of a functional gRNA duplex. RNPs were assembled by diluting 1.8μL AltR Cas9 v3 and 2μL of gRNA into 16μL of duplex buffer at RT (5μM Cas9 and 5μM gRNA final).

#### Transformation

Source cells were resuspended from a plate into 100μL TAP medium^9^ and then spread on 100mm plates with TAP in 1.5% Agar and grown in a 14h light/10h dark cycle (lights on at 8:00) for 3–4 days until a lawn formed. Resulting cells were harvested between 9:00-9:30 and resuspended in 1mL TAP in a 1.5mL tube and pelleted. All centrifugations were 600RCF for 3 min. The volume of the cell pellet was used as a proxy for cell number: The supernatant was carefully aspirated, and the pellet resuspended in 100μL TAP. This suspension was aliquoted into fresh 1.5mL tubes to result in cell pellets of 10μL (∼ $2\cdot 10^{7}$) at one tube per two different designs (integrations into one source line) and the rest of the cells discarded. Each tube was treated 3 times for 30–50 min with gamete autolysin to remove cell walls, where the third treatment was combined with 30 min heat shock at 40°C.^14^ Cells were then washed 3 times in 1.4mL TAPS (TAP+40mM sucrose) and finally resuspended with 80μL TAPS and re-aliquoted at 40μL per tube into fresh 1.5mL tubes (∼ $5-8\cdot 10^{6}$ cells per tube).

To each tube 4μL of RNP solution and 1.2μg of linearized dDNA was added. Cells were electroporated in 4 replicate reactions of 10μL (∼ $1-2\cdot 10^{6}$ cells) with the Neon electroporation system (Invitrogen) at 2300V for 12ms and 3 pulses. Finished reactions were pipetted directly into the wells of 24-well plates pre-filled with 1mL TAPS per well and left for recovery overnight. Finally, cells were concentrated by centrifugation and spread onto TAP agar plates with the corresponding antibiotic. The concentrations used were 7.5 μg/mL for Nourseothricin (NAT), 10μg/mL for Paromomycin (AphVIII), 50μg/mL for Blasticidin S (BSD) and 100μg/mL for Spectinomycin (AadA). Plates were incubated in constant light until colonies formed, between 3 and 7 days.

#### Cell line crossing and autolysin preparation

Cells of opposite mating types were spread in a thin film and grown on agar plates (10 × 100 cm plates each mating type) in constant light for 3–10 days and subsequently scraped off and resuspended in TAP-N medium. An incubation period of at least 10 h or overnight allowed for the cells to differentiate as gametes. Gametes of opposite mating types were then concentrated to ∼10^8^ cells/mL and mixed. The resulting reactions were plated on 1.5% TAP agar plates after 20–30 min and kept in constant light for one day. Plates were then wrapped in aluminum foil and kept in the dark for 5 days to allow for zygospore maturation. Finally, plates were unwrapped, scraped with a razor blade and extensively washed with TAP medium to remove unwanted vegetative cells and put in constant light until cells hatched.

For autolysin preparation 10-20 × 10cm plates of each CC-620 and CC-621 cells were grown as described above. Cells were then scraped off and resuspended individually into TAP-N and washed once in TAP-N. After 10h cells were periodically checked by mixing small aliquots until excellent mating was observed. Finally, the two suspensions were mixed, left standing for 25 min and cells removed by centrifugation at 1400 RCF for 5 min. The supernatant was filtered through a 0.2μm filter and frozen at −20°C until needed.

#### FACS sorting

All cells were sorted in a Sony MA900 sorter. The instrument was run after auto-calibration with an LE-C3210 sorting chip, featuring a 100μm nozzle. Cells were loaded and the machine equilibrated until a steady flow of ∼100–1000 cells/s was reached. Sorting was then initiated into a 96 well plate filled with 200μL of TAP per well in “single cell” mode with a count of 1 per well.

#### Screening qPCR and sequencing

Colonies from antibiotic plates were picked into 96 well plates and kept in constant light until the wells turned green. 10μL of each well were subsequently mixed with 10μL of Lucigen QuickExtract buffer and heated to 65°C for 6 min, then 95°C for 2 min qPCR plates were filled with reactions consisting of 3μL Invitrogen Platinum 2 HS mastermix, 0.15μL of 10μM primer mix targeting the desired insert, 0.5μL template from the previous step, 0.3μL EvaGreen Plus and 2.05μL of 2M Betaine. Plates were cycled in a Roche Lightcycler 96 according to the manufacturer’s recommendations. Curves were analyzed for quantification cycle Cq and high resolution melting curves by LightCycler software.

The insert of promising wells was amplified by PCR, using Invitrogen SuperFi2 Master Mix with final 1M Betaine in the reaction. PCR products were sized by 1.2% agarose gel electrophoresis in 10mM lithium-acetate-borate buffer, stained with Biotinum GelGreen and visualised on a Typhoon FLA 9500 imager. Finally, PCR products were column purified and sent for Sanger sequencing.

#### DNA gel electrophoresis

DNA gels were cast at 1.2% agarose lithium acetate borate (LAB) buffer (10mM lithium acetate, 10mM boric acid, pH 7.5) with the addition of 1x final GelGreen (Biotinum) dye. Samples were loaded in NEB purple loading dye with 10x final GelGreen dye. Finally gels were run at 16V/cm in LAB buffer and imaged on a Typhoon 9500 imager using the Cy2 laser/filter set.

#### Whole genome sequencing

50mL of dense liquid culture was pelleted by centrifugation at 1200 RCF for 10 min, followed by 4 steps of extraction with phenol-chloroform-isoamyl alcohol (25:24:1). DNA was precipitated by addition of 2 volumes of cold ethanol, pelleted at 16kRCF for 5 min, then air dried and resuspended in 200 μL TE overnight.

To identify knock-in locations and potential off-target effects, variants to the CC1690 were called based on WGS illumina reads (range from 2.6Gb to 3.8Gb–22X - 35X coverage per sample).

To find insert locations, paired-end raw sequencing reads were mapped against the insert sequences using bwa-mem (v0.7.17) with arguments -O 60 -B 50 -E 10 -L 100 -c 2 -U 50 -d 200 -w 1 and those reads with overlap to the insert sequences were then mapped to the CC1690 reference genome (NCBI Accession: GCA_013389655.1) using bwa-mem with the same parameters as above. Mapping locations of these insert-containing reads revealed the correct incorporation of all expected sites and off-target insertions in the double knock-out and *FAP256-CL7* lines (Figure S4).

To find SNPs and small indels potentially arising via off-target effects, the following pipeline was followed: Adapters were trimmed with trim_galore v(0.6.4) and paired reads were mapped to the CC1690 assembly from NCBI using bowtie2 (v2.4.5). Variants were called using deepvariant (v1.2.0), including only those variants tagged as homozygous (GT = ”1/1″) and passed deepvariant’s internal filters (FILTER = PASS). Variants were then only taken forward if they were within 10kb of a potential off-target site. Off-target sites were identified using blastn (v2.11.0), mapping guide sequences as query to the CC1690 genome as target with the task “blastn-short”. Variants were further filtered using the Ensembl Variant Effect Predictor (v106.1) to variants overlapping gene sequences with an IMPACT of MODERATE or HIGH. Identical variants that were also detected in the background strain were removed.

#### Optical microscopy

Phase contrast images were acquired on an Olympus BX61 with a UPlanFL N 40x/0.75 Ph2 objective in phase contrast and captured on an Imagingsource DMK 72AUC02 camera. Images were cropped and contrast adjusted in Adobe Photoshop.

Spinning disk micrographs have been acquired with an Andor IX 83 equipped with a Yokogawa CSU-W1 spinning disk at a 50μm pinhole size through an Olympus 150x/1.45 U Apo oil immersion objective on an Andor iXon Ultra 888 resulting in a pixel size of 79nm. Z-stacks were acquired using a Prior NanoScanZ piezo stage in 281 nm optical sections and subsequently deconvoluted with the spinning disk module of Huygens deconvolution. False color mapping was applied with Fiji.^34^ Kymograms were likewise produced using Fiji by means of the “Muli Kymograph” option.

#### Immunoblotting

Whole cells were boiled for 5min in LDS-PAGE loading buffer to create soluble lysates. Benzonase was added at ∼10U/mL to digest genomic DNA and reduce viscosity of lysates before loading onto 4–12% Bis-Tris NuPAGE gradient gels and run for 1h at 50mA in 1x NuPAGE MOPS buffer. Finished gels were transferred via iBlot onto PVDF membranes and stained with Revert total protein stain, dried with N_2_, then imaged on a LiCor Odyssey. Subsequently, membranes were shortly re-wet in 100% methanol and transferred to PBS. Immunostaining was performed with anti-IFT46 antibody 600 and LiCor CW800 goat anti-rabbit secondary antibodies using the iBind Flex FD solution kit and iBind system. Finished membranes were washed another 10 min in TBS-T before drying and re-imaging at 800 nm.

### Quantification and statistical analysis

All qPCR data was analyzed using Roche Lightcycler software. Data was plotted in Python 3.11 using matplotlib 3.5.3, pandas 1.5.3 and numpy 1.24. Chlamydomonas colonies were counted on photographs of plates using OpenCFU.^35^ Colony counts are expressed as mean ± S.E.M. Swimming or non-motile phenotypes of cells in well-plates were quantified by optical microscopy and manual counting.

## Data Availability

• All data reported in this paper will be shared by the lead contact upon request. • This paper does not report original code. • Any additional information required to reanalyze the data reported in this paper is available from the lead contact upon request.

## Supplemental information

Document S1. Figures S1–S4

Table S1. Comparison of selected reported efficiencies (ratio of positive colonies to total colonies) for landmark works on CRISPR-Cas-based genome editing of *Chlamydomonas*, related to introduction and Figure 1 Calculated efficiencies treat each targeted gene as one data point. Non-optimized experiments and experiments that directly select for a mutation were excluded. Errors are SEM between different targeted genes.

Data S1. Genomic regions of all mutant lines produced in the scope of this work, as well as vector maps of the plasmids and guide sequences used in the mutagenesis process, related to Figures 1, 2, and 4

Data S2. Original electrophoresis and blot images, related to Figures 2 and 4

Document S2. Article plus supplemental information
