# Supplementary material for: Efficient precision editing of endogenous Chlamydomonas reinhardtii genes with CRISPR-Cas
Source: Cell Rep Methods. 2023 Aug 22;3(8):100562. doi: 10.1016/j.crmeth.2023.100562 (PMC10475843; doi:10.1016/j.crmeth.2023.100562)
Supplement: Document S1. Figures S1–S4 [file mmc1.pdf]

Cell Reports Methods, Volume *3*

Supplemental information

Efficient precision editing of endogenous *Chlamydomonas reinhardtii* genes with CRISPR-Cas

Adrian Pascal Nievergelt, Dennis Ray Diener, Aliona Bogdanova, Thomas Brown, and Gaia Pigino

Supplementary Information

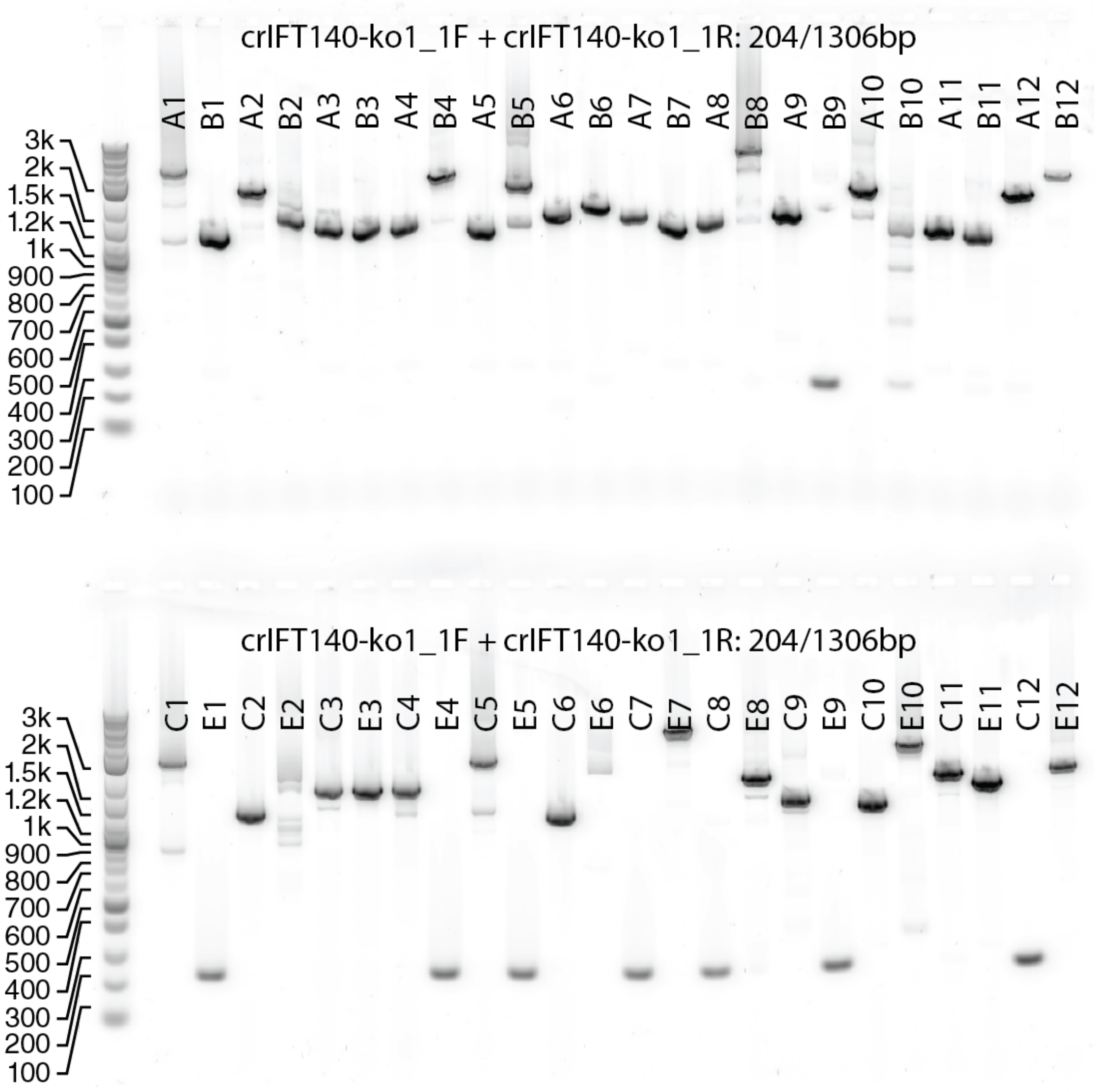

Figure S1: Annotated agarose gel electrophoresis of PCR products showing band upshifts due to insertion of a knock-out cassette in the IFT140 gene (Cre08.g362650). 8/96 bands show a wild-type band at 204bp, while the rest exhibit a variety of insertion sizes with a median around 1306bp expected for correct homology directed insertion. Related to Figure 1.

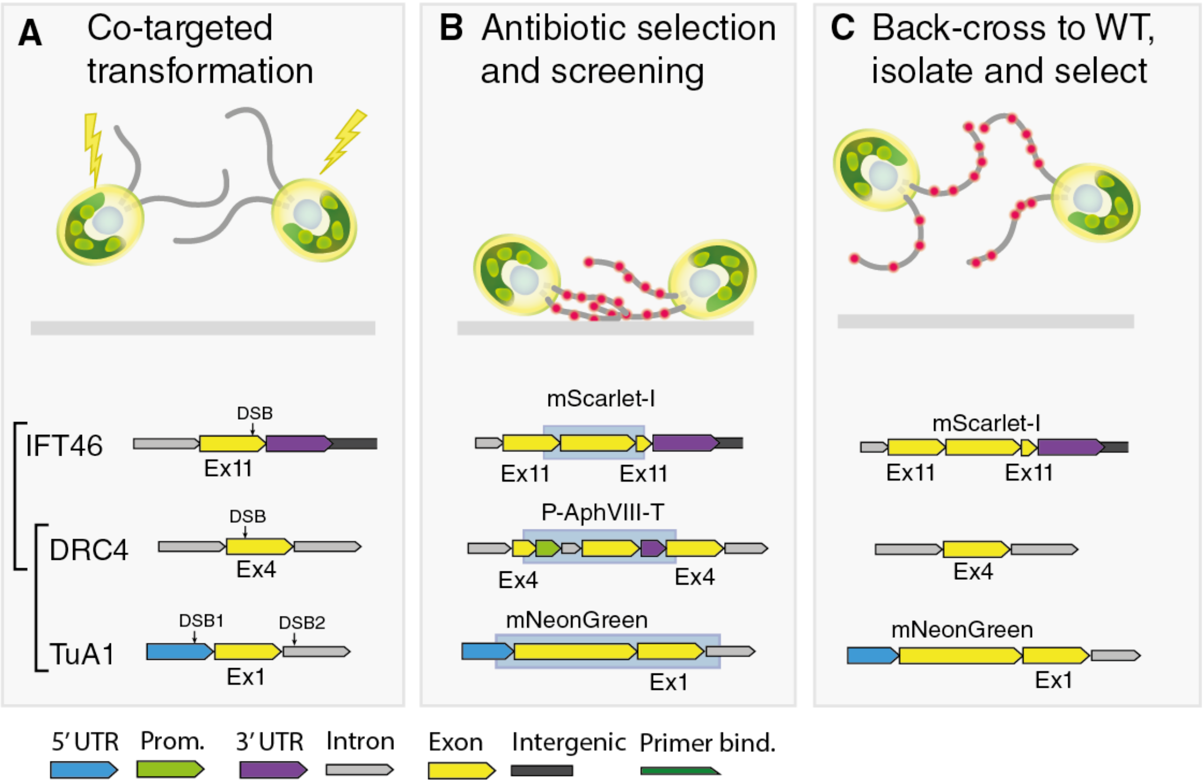

Figure S2: Co-targeting workflow for generating mutants free of selectable markers. **A)** Cells are co-transformed with a primary donor (mScarletI to IFT46-cTer / mNeonGreen to TuA1-nTer) and a secondary dominant marker to DRC4 as well as corresponding Cas9 RNPs causing necessary double stranded breaks (DSB). B) Transformed cells are selected on antibiotics plates and screened for the primary insertion. Correct clones are paralyzed, antibiotic resistant and show fluorescence of the target protein. Insertions are highlighted by blue boxes. **C)** Paralyzed primary clones are back-crossed to a wild-type line and selected for wild-type DRC4 while retaining the wanted primary insertion. Final cells are motile and show fluorescence at the target protein. Related to Figure 2.

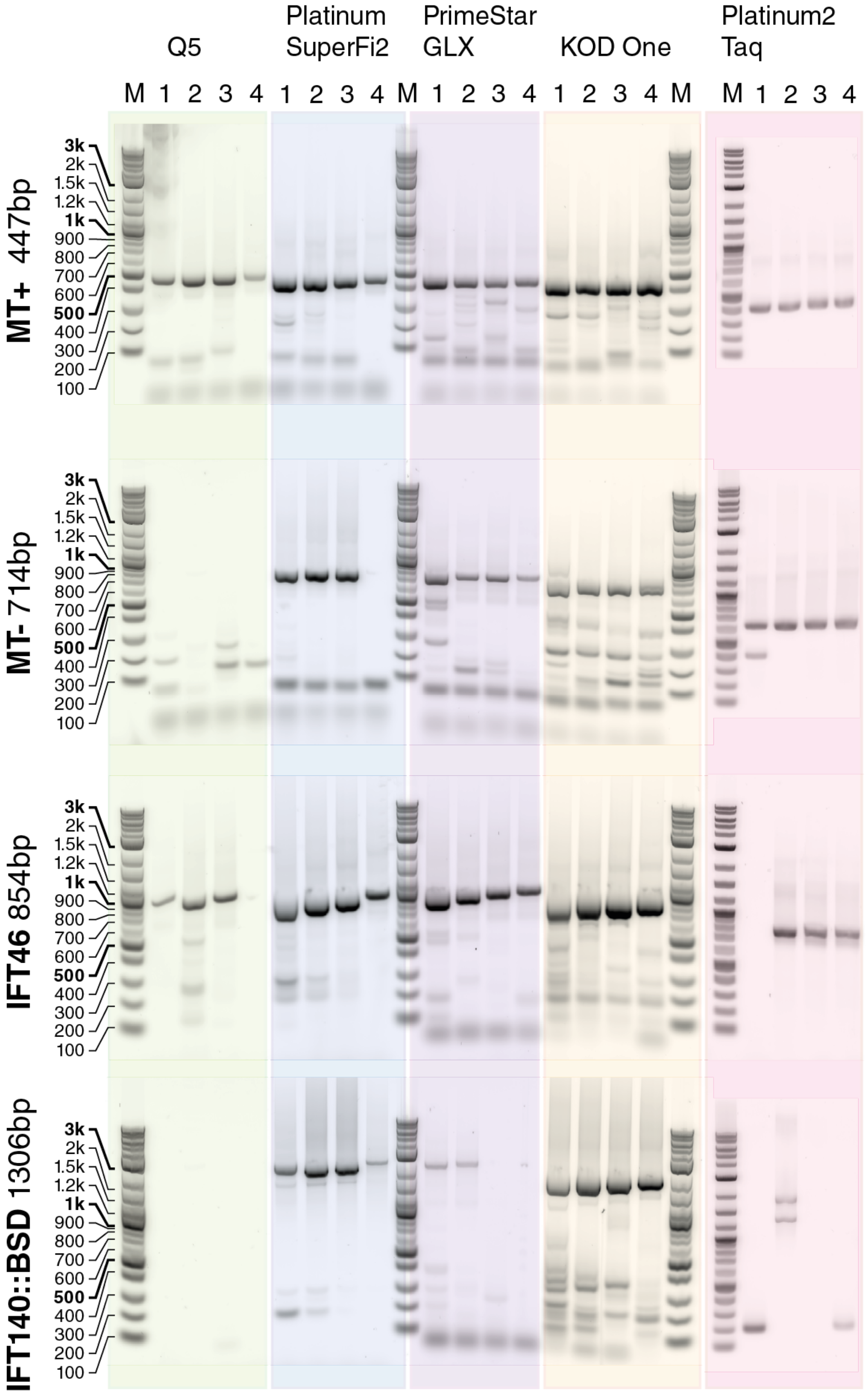

Figure S3: Agarose gel electrophoresis of different qPCR amplicons for selected polymerases (NEB Q5, Invitrogen Platinum SuperFi2, Takara PrimeStar GLX, Toyobo KOD One and Invitrogen Platinum 2 Taq) and conditions (1: As is, 2: 0.8M betaine, 3: 1.6M betaine, 4: 0.8M betaine + 0.81M propylene glycol). Related to Figure 4.

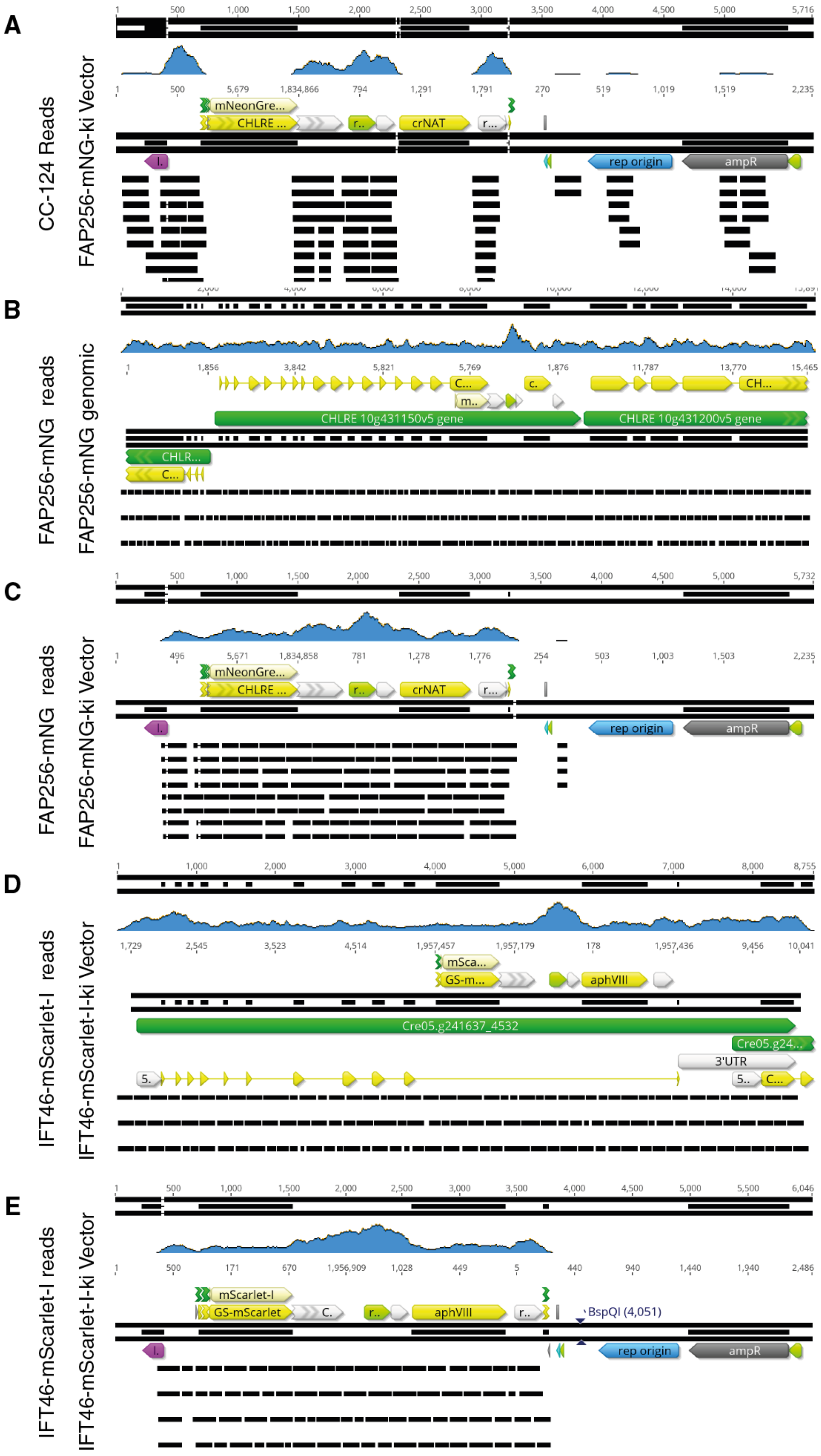

Figure S4: Whole genome alignment of CC-124, FAP256-mNG and IFT46-mScarletI against genomic design. A) CC-124 sequencing against the FAP256-mNG-ki donor vector has expected coverage against rbcS2 elements and the lacZ containing fragment of the backbone. **B)** Genomic alignment showing error-free integration

of FAP256-mNG. **C)** FAP256-mNG aligned against the donor FAP256-mNG-ki vector showing absence of non-casette integration. **D)** Genomic alignment showing error-free integration of FAP256-mScarlet-I. E) IFT46-mScarlet-I aligned against the donor IFT46-mScarlet-I-ki vector showing absence of non-cassette integration. Related to Figure 4.
