# Supplementary figures and images for: Efficient precision editing of endogenous Chlamydomonas reinhardtii genes with CRISPR-Cas
Source: Cell Rep Methods. 2023 Aug 22;3(8):100562. doi: 10.1016/j.crmeth.2023.100562 (PMC10475843; doi:10.1016/j.crmeth.2023.100562)

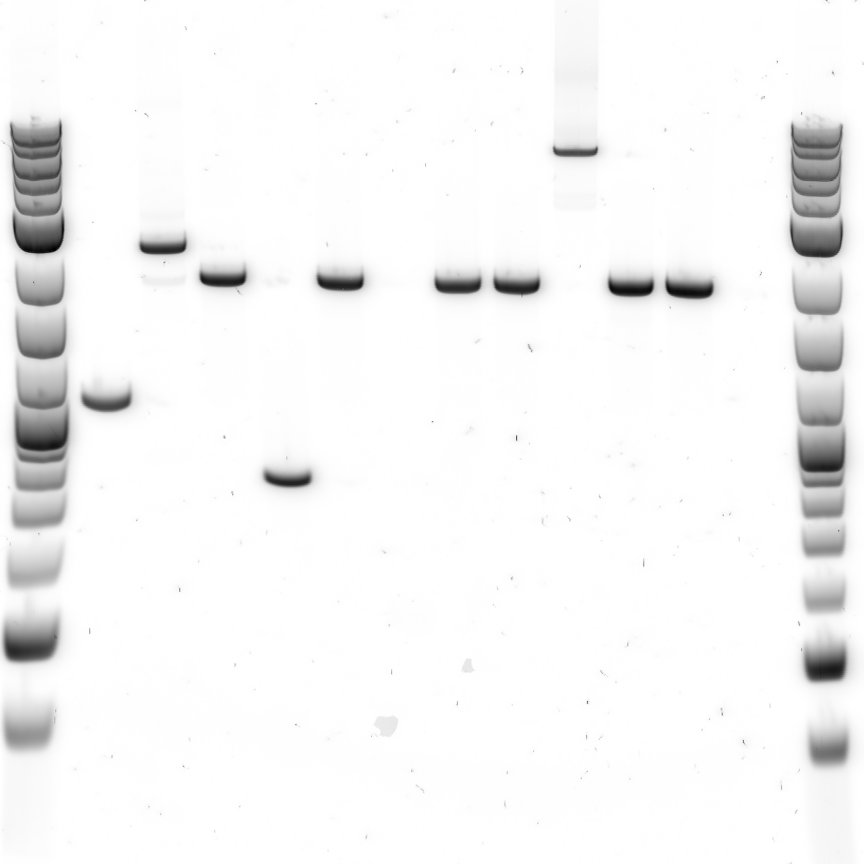

Supplement: Data S2. Original electrophoresis and blot images, related to Figures 2 and 4 [file mmc4.zip › gels_blots/2021-11-15_FAP256-mNeonGreen_SF2-[Cy2]-1-bgc.png]

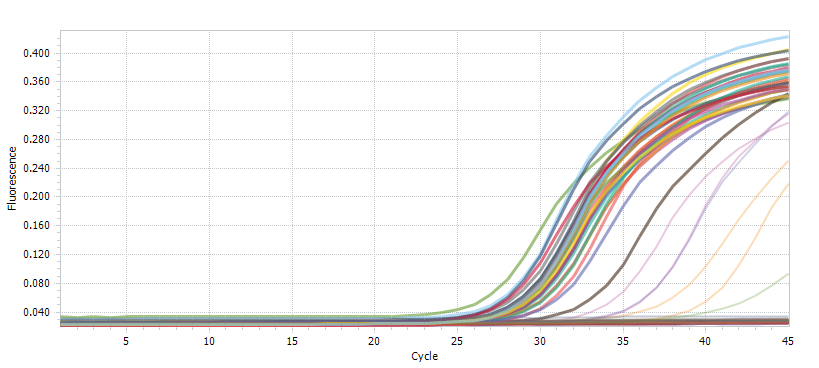

Supplement: Data S2. Original electrophoresis and blot images, related to Figures 2 and 4 [file mmc4.zip › gels_blots/Amplification_curves_FAP256-mNG.png]

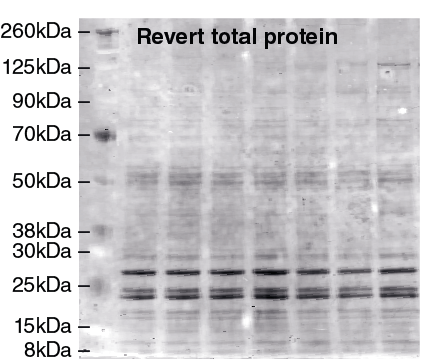

Supplement: Data S2. Original electrophoresis and blot images, related to Figures 2 and 4 [file mmc4.zip › gels_blots/sfig_western_tps.png]
